# Increased oxidative stress in shoe industry workers with low-level exposure to a mixture of volatile organic compounds

**DOI:** 10.2478/aiht-2024-75-3804

**Published:** 2024-03-29

**Authors:** Nina Umićević, Jelena Kotur-Stevuljević, Katarina Baralić, Danijela Đukić-Ćosić, Evica Antonijević Miljaković, Aleksandra Buha Đorđević, Marijana Ćurčić, Zorica Bulat, Biljana Antonijević

**Affiliations:** University of Banja Luka Faculty of Medicine, Department of Toxicology, Banja Luka, Bosnia and Herzegovina; University of Belgrade Faculty of Pharmacy, Department of Toxicology “Akademik Danilo Soldatović”, Belgrade, Serbia; University of Belgrade Faculty of Pharmacy, Department of Medical Biochemistry, Belgrade, Serbia

**Keywords:** antioxidative defence parameters, occupational exposure, trace metals, VOC mixture, metali u tragovima, parametri antioksidacijske obrane, profesionalna izloženost, VOC

## Abstract

This study aimed to assess the redox status and trace metal levels in 49 shoe industry workers (11 men and 38 women) occupationally exposed to a mixture of volatile organic compounds (VOCs), which includes aliphatic hydrocarbons, aromatic hydrocarbons, ketones, esters, ethers, and carboxylic acids. All measured VOCs were below the permitted occupational exposure limits. The control group included 50 unexposed participants (25 men and 25 women). The following plasma parameters were analysed: superoxide anion (O_2_^•−^), advanced oxidation protein products (AOPP), total oxidative status (TOS), prooxidant-antioxidant balance (PAB), oxidative stress index (OSI), superoxide dismutase (SOD) and paraoxonase-1 (PON1) enzyme activity, total SH group content (SHG), and total antioxidant status (TAS). Trace metal levels (copper, zinc, iron, magnesium, and manganese) were analysed in whole blood. All oxidative stress and antioxidative defence parameters were higher in the exposed workers than controls, except for PON1 activity. Higher Fe, Mg, and Zn, and lower Cu were observed in the exposed vs control men, while the exposed women had higher Fe and lower Mg, Zn, and Cu than their controls. Our findings confirm that combined exposure to a mixture of VOCs, even at permitted levels, may result in additive or synergistic adverse health effects and related disorders. This raises concern about current risk assessments, which mainly rely on the effects of individual chemicals, and calls for risk assessment approaches that can explain combined exposure to multiple chemicals.

## Abstract (hr)

Cilj istraživanja bio je procijeniti redoks status i koncentracije metala u tragovima kod 49 radnika (11 muškaraca i 38 žena) zaposlenih u industriji obuće, izloženih mješavini hlapljivih organskih spojeva (engl. *volatile organic compounds*, krat. VOC) koju su činili alifatski i aromatični ugljikovodici, ketoni, esteri, eteri i karboksilne kiseline. Sve izmjerene razine VOC bile su ispod dozvoljenih granica profesionalne izloženosti. U kontrolnoj je skupini bilo 50 neizloženih ispitanika (25 muškaraca i 25 žena). U plazmi obućarskih radnika analizirani su sljedeći parametri: superoksid anion radikal (O_2_^•−^), uznapredovali produkti oksidacije proteina (AOPP), totalni oksidacijski status (TOS), prooksidacijskoantioksidacijski balans (PAB), indeks oksidacijskoga stresa (OSI), aktivnost superoksid-dismutaze (SOD), aktivnost enzima paraoksonaze-1 (PON1), ukupni sadržaj sulfhidrilnih grupa (SHG) i totalni antioksidacijski status (TAS). Koncentracije metala u tragovima (bakar, cink, željezo, magnezij i mangan) analizirane su u punoj krvi. U usporedbi s kontrolnom skupinom, svi parametri oksidacijskoga stresa i antioksidacijske obrane u izloženih radnika bili su povišeni, osim aktivnosti PON1. Povećana koncentracija Fe, Mg i Zn te smanjena koncentracija Cu uočene su u izloženih muškaraca u usporedbi s kontrolnima, a u žena je primijećena povišena koncentracija Fe, a niža koncentracija Mg, Zn i Cu u odnosu na kontrolnu skupinu. Naši nalazi potvrđuju da kombinirano izlaganje mješavini VOC, čak i pri dozvoljenim koncentracijama, može dovesti do aditivnih ili sinergističkih štetnih učinaka na zdravlje i popratnih poremećaja. Trenutna procjena rizika, koja se uglavnom oslanja na učinke pojedinačnih kemikalija, izaziva zabrinutost i upućuje na potrebu za novim pristupima koji će objasniti kombiniranu izloženost većem broju kemikalija.

Volatile organic compounds (VOCs) are still commonly used in different industries as diluents in glues, primers, and degreasers. In the shoe industry, workers are often exposed to a range of VOCs, including aliphatic hydrocarbons (*n*-hexane, *n*-pentane, *n*-heptane), aromatic hydrocarbons (xylene, toluene, styrene, cyclohexane), ketones (acetone, methyl ethyl ketone, methyl isobutyl ketone), esters, ethers (diethyl ether, tetrahydrofuran, 1,4-dioxane), aldehydes (acetaldehyde, formaldehyde), alcohols (ethanol, isopropanol, butanol) and carboxylic acids (acetic, formic acid) (1). Occupational exposure to a diverse mixture of VOCs raises concern about risks of acute and chronic health illnesses and conditions, as VOCs are known to be neurotoxic, hepatotoxic, and haematotoxic (1,2,3).

Some studies looking into the mechanisms of VOC toxicity point to an endogenous imbalance between oxidants and antioxidants (4,5,6). The resulting oxidative stress directly increases free radical formation, indirectly affects the antioxidant defence system, leads to cell damage, and disrupts cell signalling and regulation of gene expression through redox-sensitive mechanisms. To fight oxidative stress, living organisms have developed defence mechanisms that involve capturing free radicals and inhibiting the formation of chelating metal ions that catalyse free radical reactions (7, 8). The main antioxidant defences include glutathione (GSH), catalase, and superoxide dismutase (SOD) (9).

In addition, some trace metals such as copper (Cu), zinc (Zn), iron (Fe), magnesium (Mg), and manganese (Mn) protect cells from damage by acting as cofactors in antioxidant reactions, and their deficiency can impair the overall defence system, increase oxygen radicals above normal physiological levels, and lead to pathological conditions (8, 9). Furthermore, almost no antioxidant enzymatic activity is possible without ion equilibrium ensured by trace metals (9, 10). Cu and Zn ions enable the activity of cytoplasmic SOD. Mn is essential for the mitochondrial type of this enzyme, Fe ions are an integral part of catalase, and Mg, as a mitochondrial antioxidant and a cofactor, plays a role in GSH production and can regulate the activity of SOD and catalase.

However, toxic effects from combined occupational exposure are often higher than exposure to individual compounds, even if they are below the occupational exposure limits (11,12,13,14). An occupational exposure limit indicates the highest permissible level of exposure to a single component by an employee during an eight-hour shift in a 40-hour work week without incurring the risk of adverse health effects (15). While the adverse health effects of exposure to single solvents above these limits are well-known, there are still uncertainties regarding the mechanisms triggered in the human body by combined exposure to low concentrations of different solvents. Our previous study (16) suggests that combined occupational exposure to organic solvents, even at low concentrations, may lead to hepatotoxicity.

Therefore, this study aimed to take a step further and investigate redox status parameters and trace metal levels among shoe industry workers occupationally exposed to a mixture of VOCs below permitted thresholds to determine the mechanistic aspects of their action.

## PARTICIPANTS AND METHODS

### Study population

In order to select the study participants, we invited 80 shoe industry workers in Bosnia and Herzegovina who had been occupationally exposed to VOCs. They were interviewed to obtain their basic anamnestic data, and their medical records were checked to exclude those who reported smoking, drinking alcohol, taking medicines, and those who had abnormal liver function tests or a history of liver disease. The study eventually included 11 male and 38 female shoe industry workers who had at least one year of work experience and were occupationally exposed to a mixture of low-level VOCs. The control group consisted of 25 healthy male and 25 healthy female workers who were not occupationally exposed to VOCs. All participants signed an informed consent to participate, and the study protocol was approved by the Biomedical Research Ethics Committee of the University of Banja Luka Faculty of Medicine, Bosnia and Herzegovina (approval no. 18/4.140/21) and the Ethics Committee for Biomedical Research of the University of Belgrade Faculty of Pharmacy, Serbia (approval no. 249/1). The study followed the ethical guidelines outlined in the Declaration of Helsinki.

We studied the effects of VOCs in workers employed in two production units. Production unit A had all male workers exposed to a combination of acetaldehyde, acetic acid, acetone, butanol, cyclohexane, ethanol, formaldehyde, formic acid, isopropanol, methyl ethyl ketone, methyl isobutyl ketone, styrene, and toluene. Production unit B had all female workers exposed to a combination of acetaldehyde, acetic acid, acetone, cyclohexane, dimethyl disulphide, dimethyl sulphide, ethanol, formic acid, isopropanol, methyl ethyl ketone, methyl isobutyl ketone, *m*-xylene, *n*-heptane, *n*-hexane, *o*-xylene, and toluene.

VOC levels measured in the air samples in both production units have been published in our previous article (16). They ranged from 0.15 mg/m^3^ (acetic acid) to 64.87 mg/m^3^ (acetone), while the permitted values ranged from 9 mg/m^3^ (formic acid) to 1900 mg/m^3^ (ethanol). In other words, the measured VOC levels ranged from two (formaldehyde) to almost several hundred times below the permitted limits (*o*-xylene, *n*-heptane).

### Sample collection

Blood samples of approximately 4 mL were collected into heparin tubes by venipuncture at the same time, in the morning after the overnight fast at the end of the working week. They were then kept at a temperature of up to 8 °C and prepared for analysis within 5 h of collection. Trace metals were analysed in whole blood and redox status parameters were analysed in plasma, separated by centrifugation at 1000 *g* for 15 min and stored at −80 °C.

### Determination of redox status parameters

Advanced oxidation protein products (AOPP) were determined spectrophotometrically at 340 nm according to the method described by Witko-Sarsat et al. (17). Chloramine-T was used for standard curve calculation in the range of 10–100 µmol/L. AOPPs are expressed in chloramine-T equivalents. The rate of nitroblue tetrazolium reduction was used to measure the level of superoxide anion (O_2_^•−^) as described by Auclair and Voisin (18).

Total oxidative status (TOS) was measured with a spectrophotometric method developed by Erel (19) using *o*-dianisidine. The results are expressed in terms of micromolar H_2_O_2_ equivalent per litre (μmol H_2_O_2_ Eq/L).

Prooxidant-antioxidant balance (PAB) was determined with a modified PAB test using 3,3^′^,5,5′-tetramethylbenzidine as a chromogen (20) and is expressed in arbitrary Hamidi-Koliakos units (HKU), which correspond to the percentage of hydrogen peroxide in standard solution.

Plasma superoxide dismutase (SOD) activity was measured according to the method of Misra and Fridovich (21). We monitored SOD-mediated inhibition of adrenalin autooxidation to adrenochrome. One unit of SOD activity is defined as the activity that inhibits adrenalin auto-oxidation by 50 %, and results are expressed as units per litre (U/L).

Paraoxonase-1 (PON1) activity was measured through paraoxon hydrolysis into *p*-nitrophenol using the method described by Richter et al. (22). We measured the rate of hydrolysis of paraoxon by monitoring the increase in absorbance at 405 nm and 25 °C. One unit (U) of paraoxonase activity is defined as 1 µmol *p*-nitrophenol formed per minute and the activity is expressed as U/L of plasma.

Total antioxidant status (TAS) was determined using an automated method developed by Erel (23). This method is based on the discoloration of 2,2′-azinobis-(3-ethylbenzothiazoline-6-sulfonic acid)-radical cation (ABTS) by antioxidants present in plasma. The change in colour was measured using an ILab 300 Plus autoanalyser (Instrumentation Laboratory, Milano, Italy). The reaction rate was calibrated with Trolox (6-hydroxy-2,5,7,8-tetramethylchroman-2-carboxylic acid), and TAS value is expressed as mmol Trolox Eq/L.

Total sulphhydryl group (SHG) levels in plasma were measured with the method described by Ellman (24). Dinitrodithiobenzoic acid (DTNB) was used as a reagent with aliphatic thiols at pH 9.0 producing *p*-nitrophenol, which was measured with spectrophotometry at 412 nm. The results are expressed as mmol/L.

Total protein levels were measured with a routine laboratory method using the biuret reagent and results are expressed as g/L.

Oxidative stress index (OSI) was calculated as the ratio between TOS and TAS parameters, as follows:

OSI (arbitrary unit) = TOS (μmol H_2_O_2_ Eq/L)/TAS (μmol Trolox Eq/L)×100 (25, 26).

### Measurement of trace metal levels

Blood samples (1 mL) were processed by microwave-assisted digestion (Milestone, Start D SK-10 T, Milestone Srl, Sorisole, Italy) with 1 mL of H_2_O_2_ (30 %) and 7 mL of HNO_3_ (68 %) using the following program: 1) heating to 180 °C for 15 min, 2) maintaining the temperature at 180 °C for another 15 min, and 3) ventilating the system and cooling for 30 min.

After digestion, all samples were restored to 25 mL volume with deionised water. Levels of Cu, Zn, Mn, Mg, and Fe were determined with inductively coupled plasma – optical emission spectrometry (ICP-OES) (Optima 8000, Perkin Elmer, Waltham, MA, USA). The elements were quantified using the external standard method, while calibration was performed using the ICP multielement standard solution IV (1000 mg/L) in diluted nitric acid (Merck, Darmstadt, Germany). The accuracy of analyses was validated with standard reference material Seronorm™ for trace elements in whole blood. The limit of detection for Fe, Mg, Zn, Cu, and Mn was 0.1, 0.04, 0.2, 0.4, and 0.1 µg/L, respectively. The obtained recovery values ranged from 99.2 % to 99.9 %.

### Statistical analysis

Statistical analysis was run on SPSS version 25.0 (IBM Corp, Armonk, NY, USA). As the variables were not distributed normally, which was determined with the Shapiro-Wilk test, comparisons between the groups were made with the Mann-Whitney *U* test, and all data expressed as medians and interquartile ranges. For possible correlations between redox status parameters and trace elements we used Spearman’s correlation coefficient. Differences at p<0.05 were considered statistically significant.

## RESULTS

The exposed and control groups did not significantly differ in age and BMI (Table 1).

**Table 1:** Participant age and BMI by sex, expressed as median and IQR

	**Exposed men (Production unit A) (N=11)**	**Control men (N=25)**	**Exposed women (Production unit B) (N=38)**	**Control women (N=25)**
Median age (years)	35 (29–37)	32 (27.5–39.5)	39 (31.75–45.25)	34 (27–40)
Median BMI (kg/m^2^)	26.90 (25.90–31.00)	24.22 (22.40–26.21)	25.75 (22.70–27.77)	24.00 (22.25–25.90)

BMI – body mass index; IQR – interquartile range (25^th^ to 75^th^ percentile)

All oxidative stress and antioxidative defence parameters were significantly higher in female workers than their controls, except for PON1, which was significantly lower. In male workers the pattern was similar, except that differences from controls were not significant for AOPP, PON1, and TAS (Table 2).

**Table 2:** Plasma redox status parameters and blood trace metal levels in shoe factory workers exposed to a mixture of volatile organic compounds vs controls by sex (means and interquartile ranges)

**Men**				**Women**		
**Parameter**	**Exposed workers (N=11)**	**Control (N=25)**	**p-value**	**Exposed workers (N=38)**	**Control (N=25)**	**p-value**
**AOPP** (µmol/L)	22.20 (19.10–38.00)	20.50 (17.53–23.06)	0.210	24.85 (21.90–26.93)	19.08 (16.37–22.69)	**<0.001**
O_2_^•−^ (mmol NBT/min	91.00 (56.00–132.00)	29.00 820.50–34.50)	**<0.001**	199.00 (135.50–263.00)	29.00 (25.50–38.50)	**<0.001**
**TOS** (µmol/L)	17.50 (14.90–21.00)	8.50 (8.10–10.05)	**<0.001**	18.55 (17.08–19.80)	8.70 (7.90–12.00)	**<0.001**
**PAB** (HKU)	115.07 (89.33–136.20)	85.38 (70.63–91.27)	**<0.001**	175.70 (159.15–195.92)	88.59 (68.37–94.94)	**<0.001**
**SOD** (U/L)	133.0 (78.00–142.00)	22.00 (15.50–43.00)	**<0.001**	139.50 (105.75–146.00)	33.00 (21.00–41.50)	**<0.001**
**PON1** (U/L)	230.00 (93.00–444.00)	347.00(205.50–435.50)	0.233	217.50 (72.75–326.75)	393.00(276.50–489.50)	**<0.001**
**TAS** (µmol/L)	1212.00 (1139.00–1261.00)	976.00 (779.00–1292.00)	0.071	996.50 (905.25–1159.00)	878.00 (760.00–1003.50)	**0.008**
**SHG** (mmol/L)	0.66 (0.63–0.69)	0.56 (0.45–0.63)	**0.001**	0.63 (0.60–0.67)	0.54 (0.50–0.59)	**<0.001**
**Total proteins** (g/L)	85.60 (82.00–89.00)	67.60 (65.50–69.20)	**<0.001**	94.30 (88.70–101.85)	67.60 (64.30–69.75)	**<0.001**
**OSI** (AU)	1.38 (1.31–1.82)	0.85 (0.68–1.13)	**<0.001**	1.84 (1.57–2.04)	0.99 (0.77–1.48)	**<0.001**
**Fe** (mg/L)	630.00 (530.40–691.20)	544.72 (527.82–564.32)	0.057	467.20 (360.40–599.40)	99.22 (77.75–117.07)	**<0.001**
**Mg** (mg/L)	46.48 (44.40–51.60)	33.91 (32.14–36.43)	**<0.001**	36.72 (29.89–41.18)	39.67 (32.83–48.70)	**0.039**
**Zn** (mg/L)	6.96 (6.08–8.52)	5.27 (4.26–5.80)	**0.001**	5.70 (2.95–7.47)	6.98 (5.28–9.04)	**0.032**
**Cu** (mg/L)	0.64 (0.60–0.84)	0.82 (0.79–0.85)	**0.024**	0.52 (0.38–0.66)	2.28 (1.35–2.66)	**<0.001**
**Mn** (µg/L)	**<LOQ**	4.86 (4.09–7.29)		**<LOQ**	3.17 (1.90–5.78)	

p-values in bold indicate significant difference between workers and matched controls (Mann-Whitney *U* test). <LOQ – below the limit of quantification; AOPP – advanced oxidation protein products; Cu – copper; Fe – iron; Mg – magnesium; O_2_^•−^ – superoxide anion radical; OSI – oxidative stress index; PAB – prooxidant-antioxidant balance; PON1 – paraoxonase-1 enzyme activity; SHG – total SH group content; SOD – superoxide dismutase; TAS – total antioxidant status; TOS – total oxidative status; Zn – zinc

Blood Fe was significantly higher and other trace metals significantly lower in the exposed women than their controls. In men the pattern was quite different. They had significantly higher Mg and Zn and significantly lower Cu levels than their controls, while the differences in Fe were not significant, although higher in the exposed group. Mn levels in the exposed workers of both sexes were below the limit of quantification (LOQ) (Table 2).

Correlation analysis allowed a deeper insight into the relationship between redox status parameters and their relationship with trace metal levels. In male workers (Table 3), AOPP correlated with TOS, OSI, and total protein levels, SOD with employment years and O_2_^•−^, SHG with TOS and OSI, O_2_^•−^ with employment years. Fe positively correlated with Mg and Zn and negatively with Cu. Mg positively correlated with Zn, and Cu negatively with TAS. There was a positive correlation between O_2_^•−^ and SOD activity and prolonged exposure to VOC mixtures (Table 3).

**Table 3:** Spearman’s non-parametric correlation between oxidative status parameters, employment duration, and trace metal levels in male workers exposed to a mixture of volatile organic compounds in the shoe industry

	**Employment years**	**Fe (mg/L)**	**Mg (mg/L)**	**Zn (mg/L)**	**Cu (mg/L)**	**AOPP (µmol/L)**	**O_2_•^−^ (mmol NBT/min/L)**	**TOS (µmol/L)**	**PAB (HKU)**	**SOD (U/L)**	**PON1 (U/L)**	**TAS (µmol/L)**	**SHG (mmol/L)**	**Total proteins (g/L)**	**OSI (AU)**
**AOPP (**µmol/L)	0.323	−0.145	0.410	0.245	0.215		0.036	0.827^**^	−0.036	0.264	0.382	−0.545	0.418	0.755^**^	0.827^**^
**O2•**^−^ (mmol NBT/min/L)	0.790^**^	0.182	0.264	0.418	−0.174	0.036		−0.091	0.336	0.709^*^	0.291	−0.136	−0.155	0.418	−0.009
**TOS** (µmol/L)	−0.037	−0.036	0.437	0.309	0.050	0.827^**^	−0.091		−0.136	−0.091	0.282	−0.482	0.745^**^	0.733^**^	0.918^**^
**PAB** (HKU)	0.199	0.318	0.292	0.145	−0.037	−0.036	0.336	−0.136		0.445	−0.073	−0.091	−0.400	0.291	0.027
**SOD (**U/L)	0.707^*^	0.255	0.528	0.518	0.110	0.264	0.709^*^	−0.091	0.445		0.509	−0.336	−0.418	0.309	0.036
**PON1** (U/L)	0.323	−0.082	0.387	0.509	0.238	0.382	0.291	0.282	−0.073	0.509		−0.464	0.173	0.264	0.300
**TAS** (µmol/L)	−0.065	0.400	−0.387	0.009	−0.783^**^	−0.545	−0.136	−0.482	−0.091	−0.336	−0.464		−0.055	−0.318	−0.673^*^
**SHG** (mmol/L)	−0.240	0.145	0.251	0.255	−0.307	0.418	−0.155	0.745^**^	−0.400	−0.418	0.173	−0.055		0.436	0.618^**^
**Total proteins** (g/L)	0.430	0.155	0.497	0.473	−0.174	0.755^**^	0.418	0.733^**^	0.291	0.309	0.264	−0.318	0.436		0.745^**^
**OSI** (AU)	−0.028	−0.100	0.501	0.136	0.293	0.827^**^	−0.009	0.918^**^	0.027	0.036	0.300	−0.673^*^	0.618^*^	0.745^**^	
**Fe** (mg/L)	−0.092		0.661^*^	0.673^*^	−0.709^*^	−0.145	0.182	−0.036	0.381	0.255	−0.082	0.400	0.145	0.155	−0.100
**Mg** (mg/L)	−0.002	0.661^*^		0.720^*^	−0.103	0.410	0.264	0.437	0.292	0.528	0.387	−0.387	0.251	0.497	0.501
**Zn** (mg/L)	0.286	0.673^*^	0.720^*^		−0.517	0.245	0.418	0.309	0.145	0.518	0.509	0.009	0.255	0.473	0.136
**Cu** (mg/L)	−0.095	−0.709^*^	−0.103	−0.517		0.215	−0.174	0.050	−0.037	0.110	0.238	−0.783^**^	−0.307	−0.174	0.293

* p<0.05.

** p<0.01 according to Spearman’s analysis.

AOPP – advanced oxidation protein products; Cu – copper; Fe – iron; Mg – magnesium; O_2_^•−^ – superoxide anion radical; OSI – oxidative stress index; PAB – prooxidant-antioxidant balance; PON1 – paraoxonase-1 enzyme activity; SHG – total SH group content; SOD – superoxide dismutase; TAS – total antioxidant status; TOS – total oxidative status; Zn – zinc

In female workers (Table 4), O_2_^•−^ strongly positively correlated with employment years, PAB, and OSI and negatively with TAS and SHG. TOS positively correlated with SHG and OSI, PAB with OSI and Cu, SHG with TOS, PON1, and total proteins and negatively with O_2_^•−^. SOD negatively correlated with PON1. Like in male workers, Fe positively correlated with Mg and Zn and Mg with Zn. SHG levels, TAS, and SOD negatively correlated with employment years (Table 4).

**Table 4:** Spearman’s non-parametric correlation between oxidative status parameters, employment duration, and trace metal levels in female workersexposed to a mixture of volatile organic compounds in the shoe industry

	**Employment years**	**Fe mg/L)**	**Mg (mg/L)**	**Zn (mg/L)**	**Cu (mg/L)**	**AOPP (µmol/L)**	**O_2_^•−^ (mmol NBT/min/L)**	**TOS (µmol/L)**	**PAB (HKU)**	**SOD (U/L)**	**PON1 (U/L)**	**TAS (µmol/L)**	**SHG (mmol/L)**	**Total proteins (g/L)**	**OSI (AU)**
**AOPP** (µmol/L)	−0.049	0.027	0.092	−0.156	0.107		0.149	0.140	0.171	0.144	−0.283	−0.124	−0.047	0.009	0.205
**O_2_•^−^** (mmol NBT/min/L)	0.334^*^	0.076	0.060	0.059	0.199	0.149		−0.119	0.289	−0.184	0.153	−0.727^**^	−0.334^*^	−0.049	0.523^**^
**TOS** (µmol/L)	−0.013	0.178	0.201	0.005	−0.122	0.140	−0.119		−0.188	−0.089	0.156	−0.012	0.453^**^	−0.233	0.555^**^
**PAB** (HKU)	0.057	−0.064	−0.119	0.014	0.704^**^	0.171	0.289	−0.188		−0.134	0.241	0.156	−0.109	0.216	0.323^*^
**SOD (**U/L)	−0.366^*^	−0.161	−0.283	−0.186	0.026	0.144	−0.184	−0.089	−0.134		−0.390^*^	0.104	0.005	−0.008	−0.109
**PON1** (U/L)	0.145	0.126	0.120	0.099	0.183	−0.283	0.153	0.156	0.156	−0.390^*^		−0.080	0.368^*^	0.333^*^	0.207
**TAS** (µmol/L)	−0.323^*^	−0.116	−0.036	−0.133	−0.273	−0.124	−0.727^**^	−0.012	−0.522^**^	0.104	−0.080		0.280	0.162	−0.806^**^
**SHG** (mmol/L)	−0.376^**^	0.237	0.195	0.036	−0.184	−0.047	−0.371^*^	0.453^**^	−0.109	0.005	0.368^*^	0.280		0.376^*^	0.069
**Total proteins** (g/L)	−0.178	0.113	0.043	0.049	0.326^*^	0.009	−0.049	−0.233	0.216	−0.008	0.333^*^	0.162	0.376^*^		−0.179
**OSI** (AU)	0.267	0.286	0.196	0.163	0.126	0.205	0.523^**^	0.555^**^	0.323^*^	−0.109	0.207	−0.806^**^	0.069	−0.179	
**Fe** (mg/L)	0.080		0.752^**^	0.851^**^	−0.233	0.027	0.076	0.178	−0.064	−0.161	0.126	−0.116	0.237	0.113	0.286
**Mg** (mg/L)	0.065	0.752^*^		0.701^**^	−0.182	0.092	0.060	0.201	−0.119	−0.283	0.120	−0.036	0.195	0.043	0.196
**Zn** (mg/L)	0.165	0.851^*^	0.701^**^		−0.108	−0.156	0.059	0.005	0.014	−0.186	0.099	−0.133	0.036	0.049	0.163
**Cu** (mg/L)	−0.091	−0.233	−0.182	−0.108		0.107	0.199	−0.122	0.704^**^	0.026	0.183	−0.273	−0.184	0.326^*^	0.126

* p<0.05.

** p<0.01 according to Spearman’s analysis.

AOPP – advanced oxidation protein products; Cu – copper; Fe – iron; Mg – magnesium; O_2_^•−^− superoxide anion radical; OSI – oxidative stress index; PAB – prooxidant-antioxidant balance; PON1 – paraoxonase-1 enzyme activity; SHG – total SH group content; SOD – superoxide dismutase; TAS – total antioxidant status; TOS – total oxidative status; Zn – zinc

## DISCUSSION

Our findings provide new knowledge about the mixture effects of VOCs in occupationally exposed populations. The majority of earlier studies (27,28,29,30,31,32,33) investigated the toxicity of a relatively high exposure to a single chemical, while the available literature related to occupational exposure to low concentrations of a larger number of VOCs in the mixture and the associated mechanisms or their outcomes is still relatively scarce (34,35,36). Workers in this and the earlier study (16), however, were exposed to a complex mixture of VOCs within the permitted levels established by national and international regulatory bodies. Even so, our findings confirm that combined exposure to VOCs can highly affect redox parameters and trace metal levels.

In terms of oxidative stress parameters, the case in point is the threefold increase in plasma O_2_^•−^ in the exposed men and sixfold increase in the exposed women compared to their controls. Exposed workers also had increased TOS, PAB, and AOPP, whose role in oxidative stress is well known (3, 26, 37). Furthermore, the calculated values of OSI, which reflects changes in the oxidant-antioxidant equilibrium, were significantly higher in the exposed workers than controls. They also evidence the presence of enhanced continuous oxidative stress in the exposed workers and are consistent with earlier studies of the effects of occupational exposure to VOCs (37,38,39).

We should also mention some observations related to the antioxidant defence system. The values of antioxidant parameters in the exposed workers were higher than in controls, except for PON1. TAS levels in male workers point to the compensatory increase in antioxidants due to chronic stimulation by prooxidants. Similar results, i. e. increased antioxidant enzyme activities after low-level exposure to chemicals present in the paint, were previously reported by Moro et al. (6). Two other studies (35, 36) also found increased oxidative stress and high activities of antioxidant enzymes in occupationally exposed nail care technicians and industrial workers. However, some studies report significantly lower antioxidants, SOD in particular, in shoe factory and petrol station workers (5, 8, 40, 41).

Our extensive analysis of correlations between measured parameters and duration of occupational exposure indicates that in spite of increase in O_2_^•−^, the concentrations of SHG, TAS, and SOD decreased in female workers, suggesting a potential compromise in their antioxidant defence mechanisms.

Existing literature (42) also reports that SHG increases with increasing TOS, probably as an adaptive mechanism to oxidative stress. We believe that increased SHG and total protein levels in our male workers could also be a compensatory mechanism against increased oxidative stress. Finally, OSI showed a positive correlation with oxidative stress parameters (AOPP, O_2_^•−^, TOS, PAB) and a negative correlation with TAS, which indicates an imbalance favouring oxidative stress.

Trace metal levels in workers exposed to solvents were investigated in several studies. For instance, Rizk et al. (8) evaluated oxidative stress and trace metal levels in Egyptian petrol station workers to find that the significant drop in antioxidant enzyme activities and trace element (Fe, Cu, and Zn) levels after prolonged occupational exposure was associated with the ability of VOCs to deplete cellular antioxidant defences (40, 41, 43, 44, 45, 46). However, changes in trace metals levels may be attributed to different dietary patterns as well.

In our study, Mg and Zn levels increased while Cu decreased significantly in the exposed men compared to control. Fe also increased, but not significantly. In contrast, the exposed women workers showed a significant, fourfold increase in Fe and a significant drop in Mg, Zn, and Cu compared to respective control. These findings may suggest some physiological variations in response to exposure to the different mixtures of solvents to which our working men and women were exposed. Similar variations in trace metal levels in workers exposed to organic solvents have been observed previously by other researchers. Mahmood et al. (47) found significantly increased Cu and Zn levels and decreased Fe in petrol station workers following long-term exposure to petrol products. Hussein et al. (5) reported no significant differences in Cu and Zn levels between shoe factory workers and their controls. However, it is difficult to compare our results with other studies because of the differences in VOC mixtures and levels present in different working environments.

Another point of interest in our study is the relation between redox parameters and trace metal levels. Considering many complex and important roles of specific trace metals in the body, it is difficult to assess their combined impact on oxidative stress. Our findings have shown that professional exposure to VOCs results in increased blood concentration of Fe. This, however, can be interpreted in two ways. On the one hand, Fe as a component of catalase may contribute to the efficacy of antioxidant defence, but on the other, high Fe levels can cause lipid peroxidation and free radical production (48). Previous studies (16, 49, 50) have shown that excess Fe promotes oxidative stress and tissue damage, resulting in liver fibrosis, hepatocellular carcinoma, and other liver pathologies. Furthermore, elevated Fe levels are occasionally accompanied by decreased Cu and ceruloplasmin levels in some tissues. In turn, high levels of free Cu may result in decreased ferroxidase activity (9). Cu plays an important role in oxidative stress and is a cofactor of the antioxidant enzyme Cu, Zn-SOD, present in cell cytoplasm. Our statistics show that only Cu significantly correlated with redox parameters. In men, Cu negatively correlated with TAS; in women, Cu positively correlated with PAB and total proteins.

We observed a positive, but not statistically significant correlation between Mg levels and TOS. As a mitochondrial antioxidant, Mg is an essential cofactor that plays a role in GSH production by gamma-glutamyl transpeptidase. Intracellular Mg can affect mitochondrial function by altering reactive oxygen species (ROS) production and by regulating the antioxidant defence system (catalase, SOD, and GSH) (51).

Our results reveal an inverse relationship between SOD and Zn in the exposed women, although Zn is a cofactor of Cu, Zn-SOD. They also show a negative correlation between Mg and SOD. The physiological role of Zn is very complex. It prevents ROS generation through several mechanisms and increases GSH synthesis, glutathione peroxidase activity, and other detoxification mechanisms. By inducing metallothionein synthesis, Zn may decrease the availability of redox metals and their participation in the Fenton reaction. Also, Zn may inhibit the susceptibility of proteins to free radical oxidation by protecting the thiol groups. However, it does not always act as an antioxidant. High intracellular Zn levels can have prooxidant properties and increase oxidative stress (52).

As for metal-to-metal interactions, we found a strong positive correlation between Fe, Zn, and Mg, which has also been suggested in liver damage (53, 54).

One of the major limitations of our study is the small sample size, which does not allow general conclusions, especially in terms of correlations between trace metals and redox parameters. In addition, we did not measure VOC levels in body fluids, which would better reflect exposure. Moreover, associating the measured plasma redox status parameters with VOC levels or their metabolites in body fluids would not only enhance the study’s mechanistic relevance but also provide a more direct link between exposure and biological response, offering a deeper insight into the complex interplay between occupational exposure to VOCs and the observed physiological changes.

These limitations underscore the need for further research with a larger sample size and a more exhaustive study design.

## CONCLUSION

Our findings confirm that long-term combined exposure to a mixture of VOCs, even at permitted levels, may result in synergistic adverse health effects and related disorders in shoe industry workers. This raises concern about current risk assessment in occupational settings, which mainly relies on the effects of individual chemicals and calls for risk assessment approaches that can explain combined exposure to multiple chemicals.
